# Analysis of accumulation patterns and preliminary study on the condensation mechanism of proanthocyanidins in the tea plant [*Camellia sinensis*]

**DOI:** 10.1038/srep08742

**Published:** 2015-03-04

**Authors:** Xiaolan Jiang, Yajun Liu, Yahui Wu, Huarong Tan, Fei Meng, Yun sheng Wang, Mingzhuo Li, Lei Zhao, Li Liu, Yumei Qian, Liping Gao, Tao Xia

**Affiliations:** 1 State Key Laboratory of Tea Plant Biology and Utilization, Anhui Agricultural University, Hefei, Anhui, China; 2 School of Life Science, Anhui Agricultural University, Hefei, Anhui, China; 3 Biotechnology Center, Anhui Agricultural University, Hefei, Anhui, China; 4 College of Horticulture, Qingdao Agricultural University, Qingdao, Shandong, China

## Abstract

In the present study, proanthocyanidins were qualitatively and quantitatively identified using hydrolysis and thiolysis assays, NP-HPLC, HPLC-ESI-MS, MALDI-TOF-MS, ^1^H-NMR, and ^13^C-NMR techniques in different organs of tea plants. The results showed that in leaves, the tri-hydroxyl, *cis*- and galloylated flavan-3-ols were the main monomeric catechins units, and (epi)catechin was found to be the major unit of polymeric flavan-3-ols when the degree of polymerization was greater than five. In roots, the PAs were found to be abundant, and epicatechin formed the predominant extension unit of oligomeric and polymeric PAs. In order to understand the mechanism of proanthocyanidins polymerization, auto-condensation of the flavan-3-ols was investigated. The results showed that the same trimers (*m/z* 865) were detected in the extracts of tea plants and in the non-enzymatic *in vitro* assay, in weak acid as well as weak alkaline solutions at room temperature, when the substrates used were either procyanidin B2 and monomeric flavan-3-ols (epicatechin or catechin), or only procyanidin B2. This suggested that procyanidin B2 not only released carbocation as electrophilic upper units, but also could be used as nucleophilic lower units directly itself, to form the procyanidin trimer *in vitro* or *in vivo*.

Proanthocyanidins (PAs), also called condensed tannins (CTs), are an important class of structurally complex secondary metabolites and exist naturally as phenolic compounds. PAs may occur as oligomers when the degree of polymerization (DP) = 2–10, and as polymers if DP > 10^1^. Mixtures of oligomers and polymers are composed of monomeric catechins units linked mainly through C4–C8 and/or C4–C6 bonds (B-type), or through a C4–C8 bond with an additional C2-O7 ether bond (A-type) (Fig. S1)^2^. A-type PAs are less frequently encountered in nature than B-type^3^.

PAs, which have beneficial effects on human health, are found in many food products including vegetables, fruits, wines, and cereals^1^,^4^,^5^. However, there has been limited research focusing on the accumulation pattern of PAs in tea plants. Tea is rich in monomeric flavan-3-ols, especially galloylated catechins^6^. Therefore, compared to monomeric flavan-3-ols, the accumulation of PAs has been traditionally thought to be very low in tea plant. Moreover, in our recent study, galloylated catechins and PAs were found to be the dominant flavonoids in tea leaves and roots, respectively^6^. We further suggested that the galloylation and polymerization of flavan-3-ols were the main reactions occurring in tea leaves and roots, respectively (Fig. 1). Although the biochemical mechanism of galloylation of catechins to form galloylated catechins has been studied^7^, the process of polymerization of monomeric catechins into PAs still remains unclear. Comparative studies of flavonoid metabolism in tea leaves and roots will provide greater insights into the mechanisms of galloylation and polymerization or condensation, successful manipulation of monomeric catechins, and PAs biosynthesis.

The purification, identification, distribution, and biosynthesis of catechins in tea plants have been widely researched^6^,^8^,^9^; however, similar reports involving PAs in tea plants are relatively meager^3^,^10^,^11^,^12^. Several analytical techniques have been used to determine the complex structural characteristics of PAs, which include hydrolysis, thiolysis, and normal-Phase HPLC (NP-HPLC) analyses^1^,^13^,^14^. Nevertheless, the more recently used techniques include liquid chromatography combined with electrospray ionization mass spectrometry (HPLC-ESI-MS), matrix-assisted laser desorption/ionization time of flight mass spectrometry (MALDI-TOF-MS), H nuclear Magnetic Resonance (^1^H-NMR), and C nuclear Magnetic Resonance (^13^C-NMR)^3^,^5^,^15^,^16^. In the present study, PAs in different organs of tea plants were qualitatively and quantitatively analyzed using hydrolysis and thiolysis assays, NP-HPLC, HPLC-ESI-MS, MALDI-TOF-MS, ^1^H-NMR, and ^13^C-NMR techniques.

The condensation mechanism of proanthocyanidins has gained much attention since the 1970s. Haslam et al. hypothesized that the condensation mechanism of procyanidin involved two distinct units, the nucleophilic flavan-3-ols, and the electrophilic C-4 carbocation^17^,^18^. There is still a lack of understanding of the PA biosynthesis and its regulation, despite the significant progress made in comprehending the biochemistry and molecular genetics^19^, the PA transport forms^20^, and transcription regulatory mechanisms since the 21st century^21^,^22^,^23^,^24^,^25^,^26^,^27^,^28^. For PA biosynthesis mechanism, the general view perceived is that the flavan-3-ols, (+)-catechin and (−)-epicatechin, are the building blocks of proanthocyanidins; and at first, PA polymerizes into cytoplasmic vesicle-like structures and then the latter fuses with the central vacuole^29^. The biosynthesis of flavan-3-ols, such as (+)-catechin and (−)-epicatechin, first occurs on the cytosolic side of the endoplasmic reticulum (ER) surface, then the (−)-epicatechin is catalyzed and transformed into epicatechin 3′-*O*-glucoside by a glycosyltransferase^20^. Epicatechin 3′-*O*-glucoside has been proven to be a substrate for a vacuolar multidrug and toxic compound extrusion (MATE) transporter^30^. However, in the vacuoles, the mechanism involving condensation of (−)-epicatechin or epicatechin 3′-*O*-glucoside into PA oligomer *in vivo* still remains a mystery^19^.

Some biochemical and molecular genetic studies offer theories about the polymerization of flavan-3-ol [(+)-catechin and (−)-epicatechin] units to form proanthocyanidin. For example, procyanidin B3 may be obtained from the oxidative reaction of catechin, catalyzed by a tyrosinase^31^; and the A-type linkages (the C7 hydroxyl to C2) can result from polyphenol oxidase-mediated oxidation, when epicatechin is incubated with banana extracts^32^. Recently, Pourcel et al. cloned a *TT10* gene, which expressed a laccase-like enzyme that may be involved in the oxidative polymerization of flavonoids in Arabidopsis seed coat^33^ (Fig. 1). From their results, it could be concluded that the laccase-like enzyme is involved only in the polymerization of PA oligomers into polymers, because the *tt10-2* mutant contained 4.6-fold more soluble PAs (PA oligomers) than the wild-type control.

There are some reports available on the chemical oxidation of flavan-3-ols. For instance, treatment of procyanidin B1 or B2 with the oxidizing agent, 1,1-diphenyl-2-picrylhydrazyl radical, resulted in the formation of procyanidin A1 or A2^34^. The flavan-3-ols, including (+)-catechin, (−)-epicatechin, and epigallocatechin gallate, could be oxidized *in vitro* by PPO into theaflavins (TFs) and thearubigins (TRs), which exist only in processed tea, such as black tea, and not in fresh tea plants^35^,^36^,^37^.

In the present study,we find that the same procyanidin trimers (*m/z* 865) could be detected in the extracts of tea plants as well as in the non-enzymatic *in vitro* assay, when either procyanidin B2 with epicatechin (EC) or catechin (C), or only procyanidin B2 were used as a substrate in acid and alkaline solutions, respectively. This suggested that procyanidin B2 not only released the carbocation as electrophilic upper units, as expected, but also as nucleophilic lower units, to form the procyanidin trimer *in vitro* or *in vivo*. This phenomenon may contribute to a greater understanding of the PAs biosynthesis and regulatory mechanisms.

## Results

### Identification of PAs in Different Organs of Tea Plants

#### RP-LC-ESI-MS Assay

Liquid chromatography (LC) combined with electrospray ionization mass spectrometry (ESI-MS) are particularly useful for the qualitative analysis of compounds^6^,^38^,^39^. As presented in Table 1 and Fig. 2, eight monomers and 12 oligomers of PAs existing in the roots, stems, and leaves were identified through LC-TOF-MS and UPLC-QQQ-MS/MS techniques, based on various standards reported in the literature, including t_R_, λmax, ([M + H]^+^/[M − H]^−^), and major fragment ions in tea or other plants^8^,^40^,^41^,^42^,^43^. Among these compounds, eight monomers and 10 PAs oligomers were detected in leaves and stems, with high contents of monomers (including catechin, epicatechin, gallocatechin, epigallocatechin, epicatechin gallate and epigallocatechin gallate) as compared to their oligomers (Table 1, Fig. 2A and 2B). In contrast, only one monomer (epicatechin) and a few oligomers were detected in roots, which suggested that the oligomers were dimers and trimers of flavan-3-ol with di-hydroxyl groups in the B-ring (Table 1, Fig. 2C). Unfortunately, it was not possible to perform accurate qualitative or quantitative analysis of PAs with molecular weights more than 1000, including oligomers (DP = 4–10) and polymers (DP > 10).

#### Hydrolysis Assay

PAs were hydrolyzed into colored anthocyanidins in butanol-HCl solution upon heating^44^. The hydrolyzed products were analyzed by RP-HPLC at 530 nm to identify the hydroxyl groups in the B-ring of the extension units of PAs. The data showed three product peaks in leaves and stems (Fig. S2A and S2B), but only one peak was detected in roots (Fig. S2C). According to the standards, peaks 1, 2, and 3 represented delphinidin, cyanidin, and pelargonidin, respectively. This suggested that the extension units of PAs with mono-, di-, and tri- hydroxyl groups in the B-ring existed simultaneously in leaves and stems; while in roots, only the extension units with di-hydroxyl groups in the B-ring occurred. These results were consistent with the findings of LC - MS.

#### Thiolysis Assay

DP of PAs was determined by thiolysis. As shown in Table 2, the DP increased from leaves to stems and roots. In addition, the DP in different developmental stages of leaves and roots did not vary substantially. For example, the DP in annual and biennial leaves was determined as 1.03 ± 0.02 and 1.02 ± 0.03, respectively, and in fibrous and tap roots, the values were found to be 5.85 ± 0.05 and 5.41 ± 0.11, respectively. However, significant differences in the values of DP were observed in different developmental stages of stems (Table 2). Interestingly, the PAs showed a significantly higher DP in the biennial stem as compared to the annual stem. In summary, the DP of PAs increased gradually from the upper morphological region of the tea plant to the lower part. The average value of DP, obtained through thiolysis assay, explained the molecular weight distribution characteristics of PAs in different organs of a tea plant.

#### NP-HPLC Assay

Various researchers have used normal-phase-HPLC to quantify the oligomeric and polymeric PAs in foods^1^,^5^,^14^,^45^. For instance, Gu et al.^1^,^5^ in their assay project, applied NP-HPLC-MS/MS to screen for PAs in 88 different kinds of foods, and PAs up to decamers were separated according to the DP values. Polymers with DP > 10 were eluted as a single peak at the end of the chromatogram. In the current study, oligomeric and polymeric PAs in leaves, stems, and roots of tea plants were identified using the same methodology (Fig. 3A, 3B and 3C). The results showed a great variation in the composition of oligomeric and polymeric PAs between leaves and roots, while only monomers were detected in the leaves (Fig. 3A). A series of oligomeric PAs were detected, and a large peak of polymers (DP > 10) was present at the end of the chromatogram (Fig. 3C). These observations were in agreement with the results of the thiolysis assay. The oligomeric PAs with DP less than ten were separated and qualitatively analyzed by NP-HPLC.

#### MALDI-TOF-MS Assay

Matrix-assisted laser desorption/ionization time-of-flight mass spectrometry (MALDI-TOF-MS) is appropriate for the analysis of a mixture of biomacromolecules. PAs in leaves and roots were analyzed by MALDI-TOF-MS using 2,5-dihydroxybenzoic acid (DHB) as a matrix in reflection positive ion mode (Table 3, Fig. 4). As presented in Fig. 4A, a series of peaks with ions corresponding to cesium adducts [M + Cs]^+^ distributed from *m/z* 995.1264 to *m/z* 2728.4940, were reviewed in the mass spectrum of MALDI-TOF-MS. The (epi)catechin, (epi)gallocatechin, (epi)catechin gallate, or (epi)gallocatechin gallate monomers were analyzed based on mass increments of 16 Da, 32 Da, 48 Da and 152 Da with identical DP (Table 3, Fig. 4A and 4B); thereby suggesting the presence of a series of oligomers in leaves, with DP ranging from 3 to 9. Remarkably, only one obvious signal peak was observed when DP was greater than five. The cesium adducts of the peak with different DP increased at a distance of 288 Da, corresponding to the catechin/epicatechin monomer. The results indicated that the PAs with DP > 5 comprised of epicatechin or catechin monomer units in tea leaves. It was proved that the PAs in leaves mainly comprised of the B-type with C-C bond linkages. The A-type was also detected in PAs with a DP of 3, with one or two additional C2 -O7 ether bonds; for instance, at *m/z* 1179.0103, *m/z* 1181.0724, and *m/z* 1183.1965 (Table 3, Fig. 4C).

A series of peaks with the cesium adducts [M + Cs]^+^, distributed from *m/z* 999.3355 to *m/z* 3593.3321 were detected in the MALDI-TOF mass spectrum of PAs in tea roots. The distance between each adjacent ion peak was 288 Da, corresponding to one catechin/epicatechin monomer (Table 3, Fig. 4D). This suggested the presence of a series of oligomers and polymers in roots, with DP ranging from 3 to 12. The catechin and/or epicatechin monomers were the predominant structural units of PAs. Moreover, only B-type PAs with C-C bond linkage were detected in roots. However, MALDI-TOF-MS failed to detect higher polymers of PAs (DP > 12). Also, the technique could not identify the epimerization of PA units, or assist with quantitative determination of PAs.

#### ^13^C-NMR Assay

Various studies on the characterization of PAs^15^,^16^,^46^ have suggested that ^13^C-NMR spectroscopy facilitates the structural characterization of oligomeric and polymeric PAs, including the stereochemistry of the heterocyclic ring at C2–C3 position, the hydroxylation pattern of the B-ring, the structures of the chain terminating units, and the DP of PAs. The ^13^C-NMR spectrum of PAs from tea plants is portrayed in Fig. 5.

The signals at *δ* 114–115 ppm (C2′, C5′), 118–120 ppm (C6′), and 144–145 ppm (C3′, C4′) were ascribed to catechin units with di-hydroxyl groups in the B-ring, while the signals at *δ* 145–146 ppm (C3′, C5′), 132–133 ppm (C4′), and 106–107 ppm (C2′, C6′) typically denoted the presence of catechin units with tri-hydroxyl groups in the B-ring. The spectrum (Fig. 5) suggested co-existence of di- and tri-hydroxyl groups in monomeric catechins and PAs of leaves. Leaves contained 12.9% of the units with di-hydroxyl groups whereas the roots showed 100% di-hydroxyl groups in the B-ring, as determined by the area ratio of 144–145 ppm to [144–145 ppm + 145–146 ppm].

The C-2 region occurred at 75–85 ppm, with 80 ppm as the dividing line between *cis-* and *trans-* structures. The signals at 75–77 ppm were attributed to the top extension units of the C2-*cis* form. The peak at 77–79 ppm was ascribed to C2-*cis* isomer in terminal units or monomeric catechins. A chemical shift of more than 80 ppm was the characteristic signal of the C2-*trans* configuration. The monomeric catechins and the polymeric PAs could not be distinguished by C-2 region. The spectrum presented in Fig. 5, also indicated the predominance of *cis* form, irrespective of the location. According to the area ratio of C2 (75–80 ppm) to [C2 (75–80 ppm) + C2 (80–85 ppm)], leaves contained 95% *cis* structure, while the roots comprised 100% C2-*cis* isomers.

The DP could be determined from the ratio of the peak areas of [C3 top and extension units to C3 terminal and monomeric units] + 1. The shifts for C3 terminal and monomeric units, and C3 top and extension units occurred at 65.0–69.5 ppm and 69.5–75.0 ppm, respectively. As illustrated in Fig. 5, the signals for C3 occurred in leaves mainly at a concentration of 65.0–69.5 ppm, showing that the DP of PAs was 1.03. In roots, the C3 top and extension units were primarily present and the DP of PAs was estimated as 5.44. These results were also consistent with the thiolysis data.

When PAs were linked by C4–C8, the chemical shifts of C8 in extension and terminal units occurred at 106–107 ppm. The C4 in extension and terminal units occurred at 37–38 ppm. As shown in Fig. 5B, roots yielded evident signals involving extension and terminal units of C8 (106–107 ppm) and C4 (37–38 ppm), confirming the existence of C4–C8 linkage. In comparison to the roots, signals involving extension and terminal units of C8 in leaves overlapped with C2′and C6′(tri-hydroxyl groups in the B-ring), and C4a-*trans* isomer. Weak signals of extension and terminal units of C4 (Fig. 5A) prevented easy confirmation of the linkage patterns in leaves.

These results, therefore, indicated co-existence of di- and tri-hydroxyl groups in the B-ring in leaves. C2-*cis* units were most commonly found. Polyphenols included various monomeric catechins. In roots, the units consisted only of di-hydroxyl groups in the B-ring, and C2-*cis*, with a DP > 5.

#### Purification and ^1^H-NMR Characterization of PAs in Roots

PAs from roots of tea plants were purified and identified to facilitate their structural characterization with ^1^H-NMR assay. The rough extracts of PA were separated into three sections (spot A, B, and C) by GF254 silica gel plate (Fig. S3 left). RP-HPLC analysis showed that the relative shift values (RF) of spot A (0.71) and spot B (0.29) were in accordance with the standard substances epicatechin (EC) and procyanidin B2, respectively (Fig. S3A and 7B). Spot C was found to localize at the origin, which made it difficult to separate (Fig. S3C). Compared with the silica gel plate, silica gel H column improved the purification efficiency owing to the large amounts of sample used. The rough extracts of PAs were further separated into six fractions (S1 to S6) on silica gel H column and stained with Vanillin-H_2_SO_4_ and DMACA-HCl reagents (Fig. 6A). The DP of PAs increased gradually from S1 to S6 fractions via TLC and NP-HPLC (Fig. 6B and 6C). The fractions S1 and S2 were relatively simpler, and the single peak in fraction S6 suggested the existence of polymers (Fig. 6C). The S1, S2, and S6 fractions were purified by preparative HPLC.

**Figure 1:**
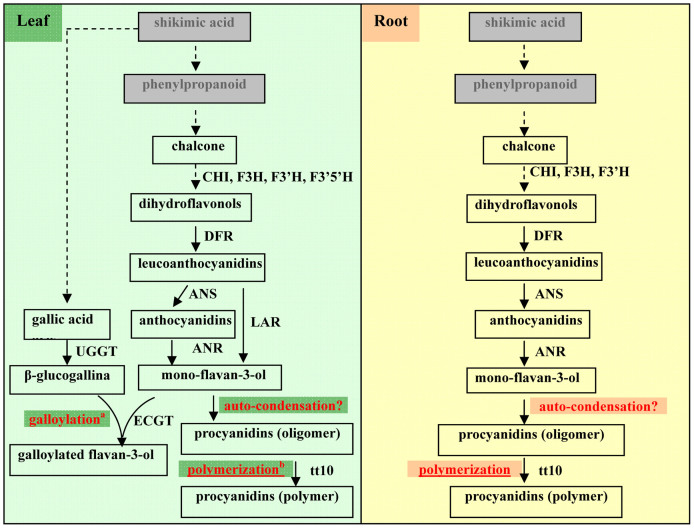
The biosynthetic pathways of flavan-3-ols and their derivatives in leaves and roots of tea plants. NOTE: The galloylation and condensation are the major terminal pathways of flavan-3-ols biosynthesis in the leaves and roots of tea plants, respectively. (a) Based on data from Liu et al. (2012); (b) From Pourcel et al. (2005).

**Figure 2:**
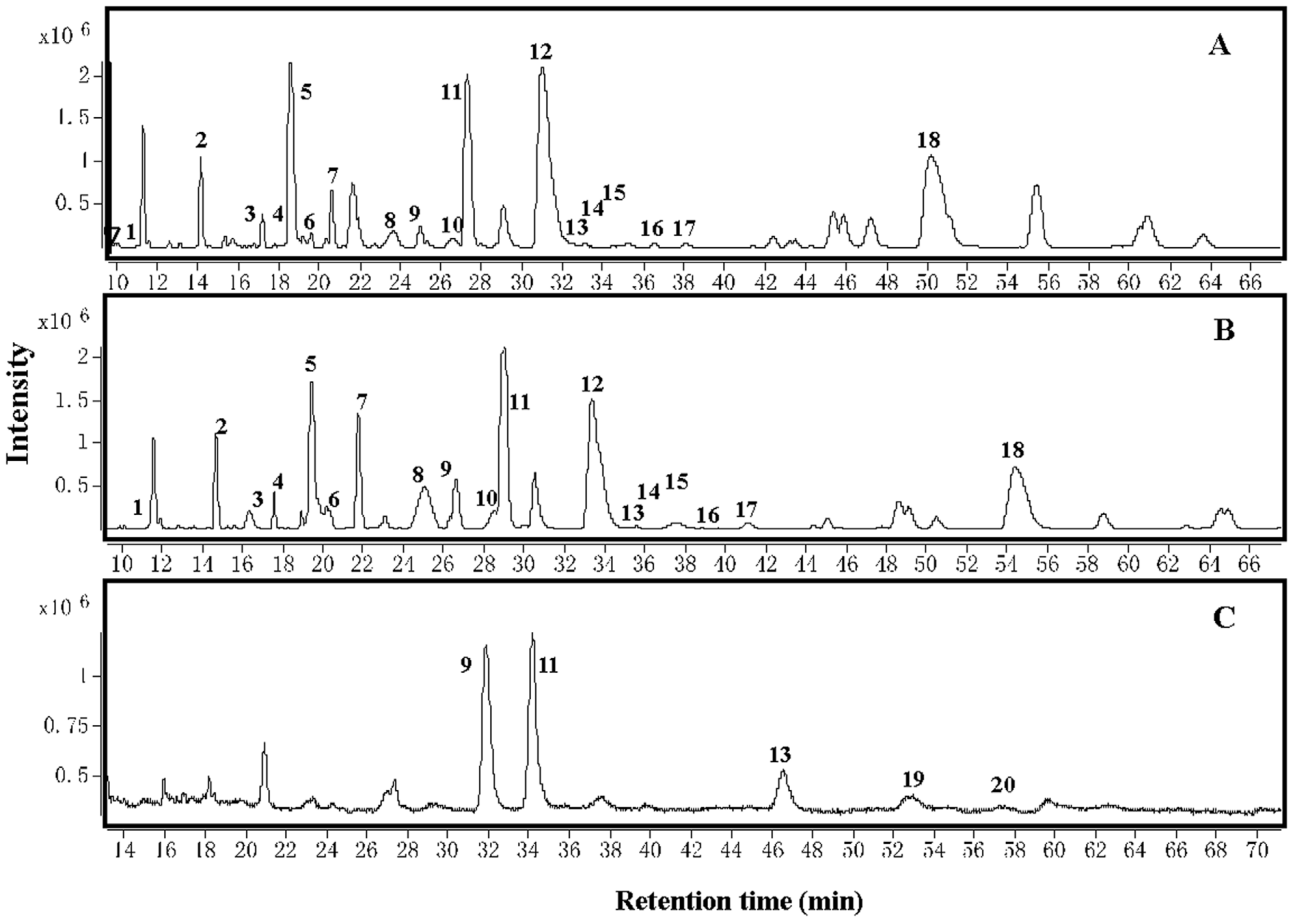
Total ion chromatogram of catechins and proanthocyanidins from leaf (A), stem (B) and root (C) extracts of tea plants. The sample preparation methods are described in the Materials and Methods. The compounds are identified and listed in Table 1.

**Figure 3:**
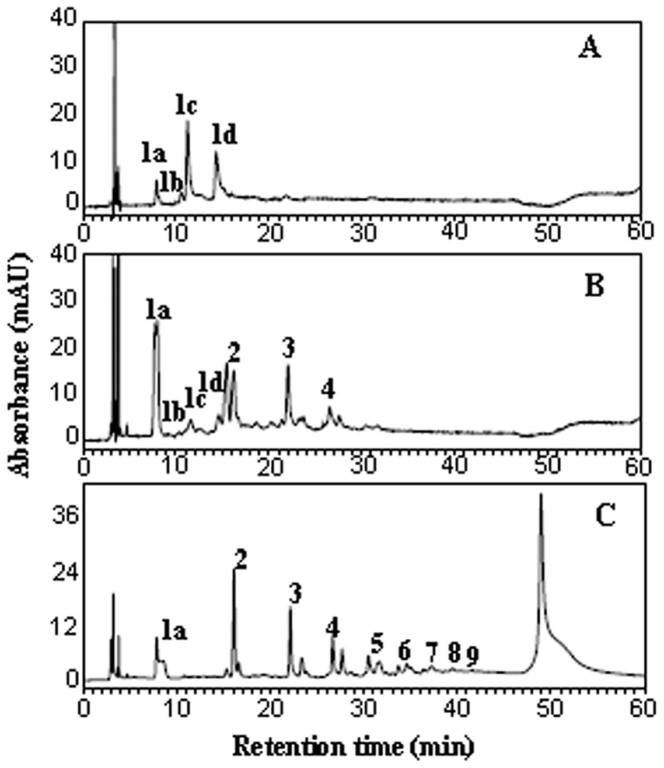
NP-HPLC analysis of catechins and proanthocyanidins from leaf (A), stem (B) and root (C) extracts of tea plant. (1a): catechin and epicatechin; (1b): gallocatechin and epigallocatechin; (1c): epicatechin gallate; (1d): epigallocatechin gallate; 2 ~ 9 represent degree of polymerization of proanthocyanidins.

**Figure 4:**
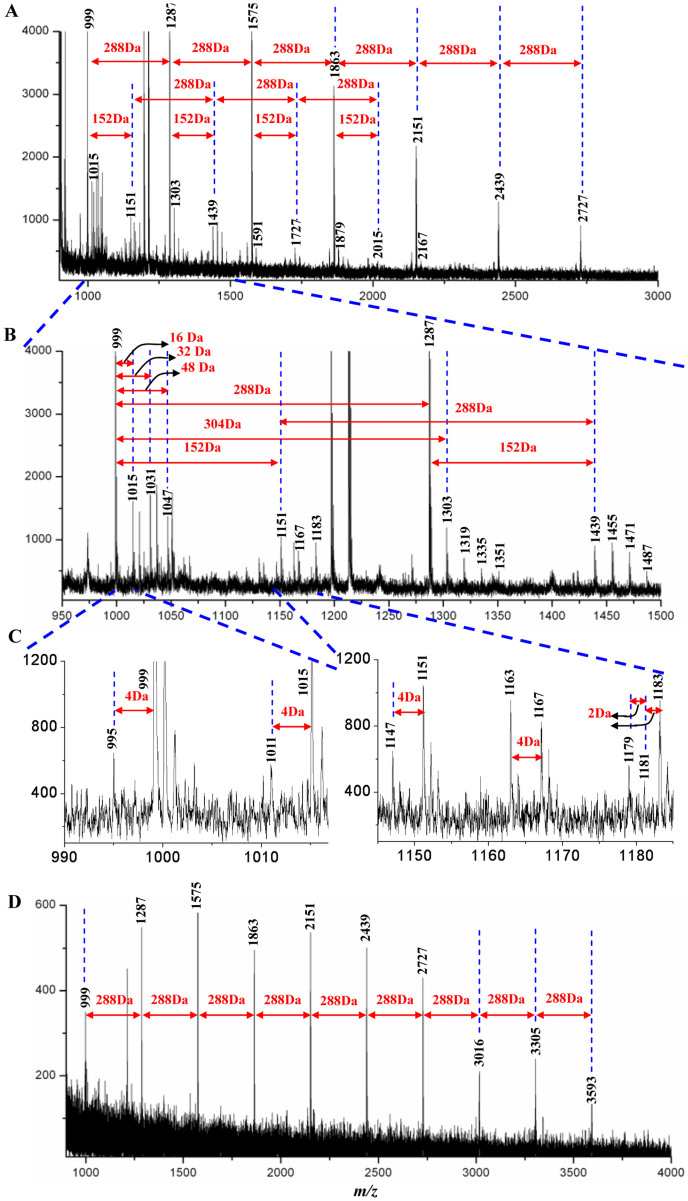
MALDI-TOF mass spectrum of proanthocyanidin extracted from leaves and roots of tea plants. (A), leaves; (B), partially amplified mass spectrum of A revealed the proanthocyanidin with DP = 3 and 4; (C), partially amplified mass spectrum of B with different linkage types; (D), roots.

**Figure 5:**
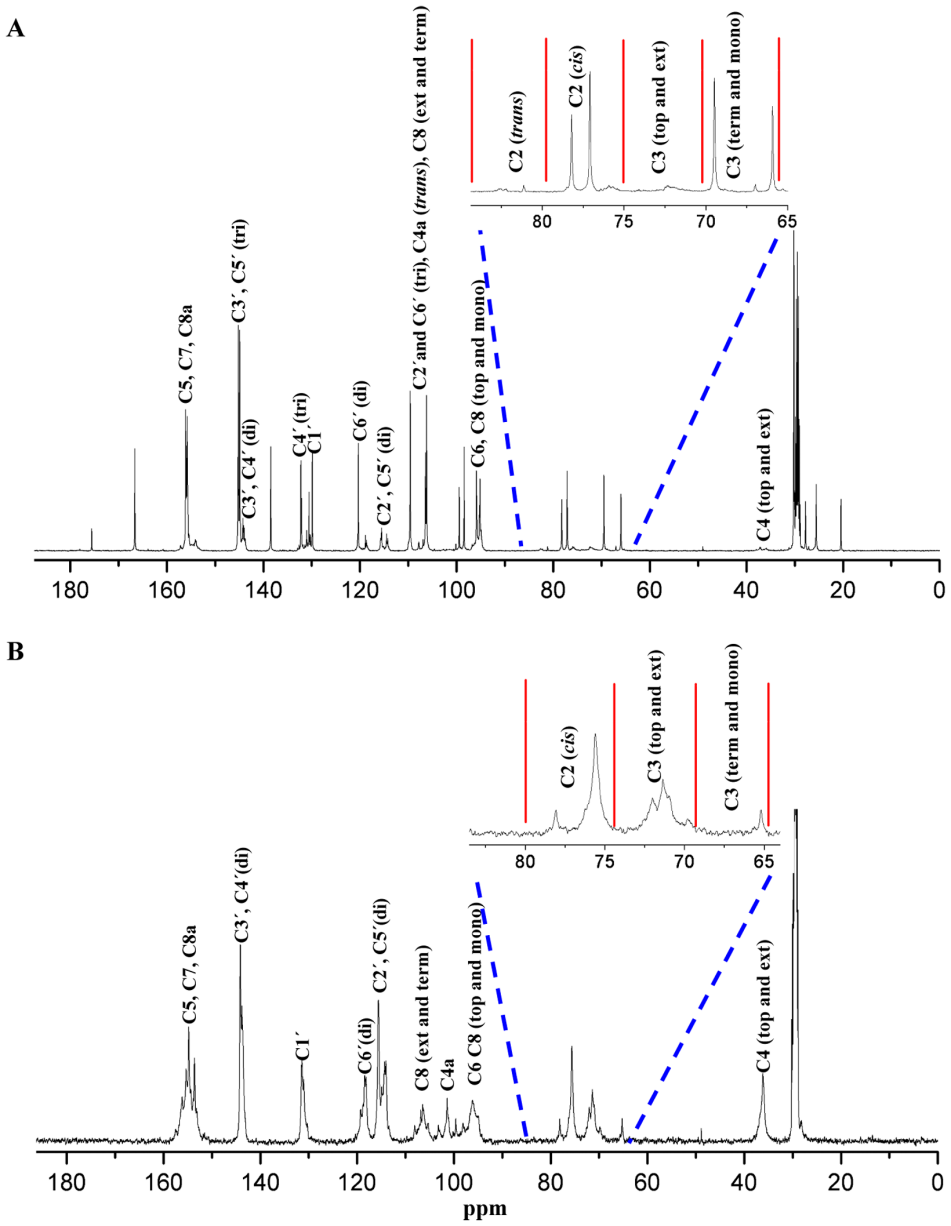
^13^C-NMR spectrum of catechins and proanthocyanidins from leaf (A) and root (B) extracts of tea plant in acetone-*d_6_*/D_2_O. term, terminal unit; ext, extension unit; top, top unit; mono, monomeric catechins; di, dihydroxyl groups in the B-ring; tri, trihydroxyl groups in the B-ring.

**Figure 6:**
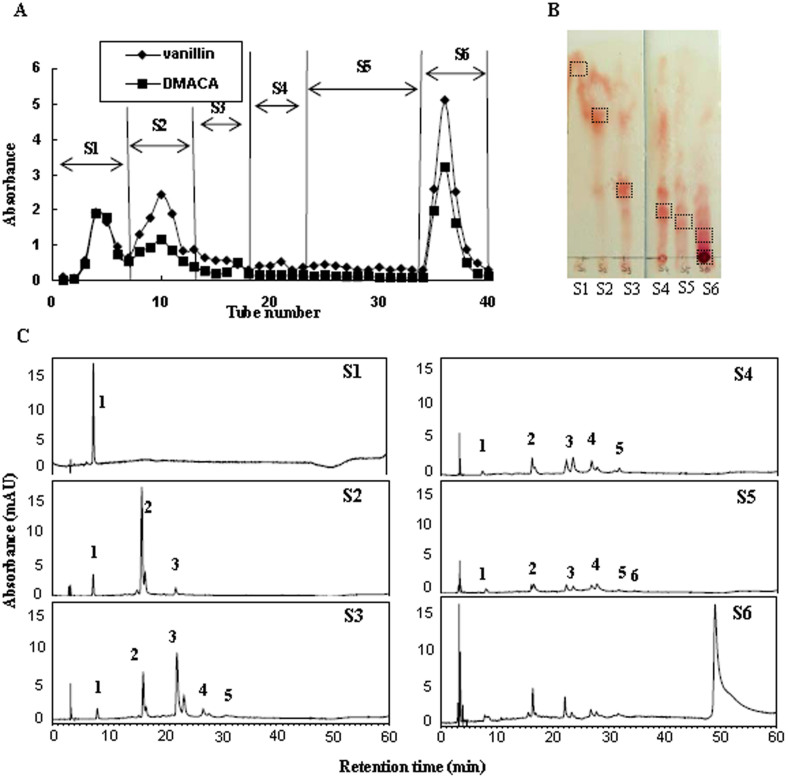
Elution effects of proanthocyanidins (PAs) purified by silica gel column. (A), Chromatography of PAs purified by silica gel column stained with Vanillin and DMACA reagents; (B), TLC analysis of different p PAs fractions purified by silica gel column; (C), NP-HPLC analysis of different PAs fractions purified by silica gel column. 1 ~ 9 represent degree of polymerization of PAs.

**Figure 7:**
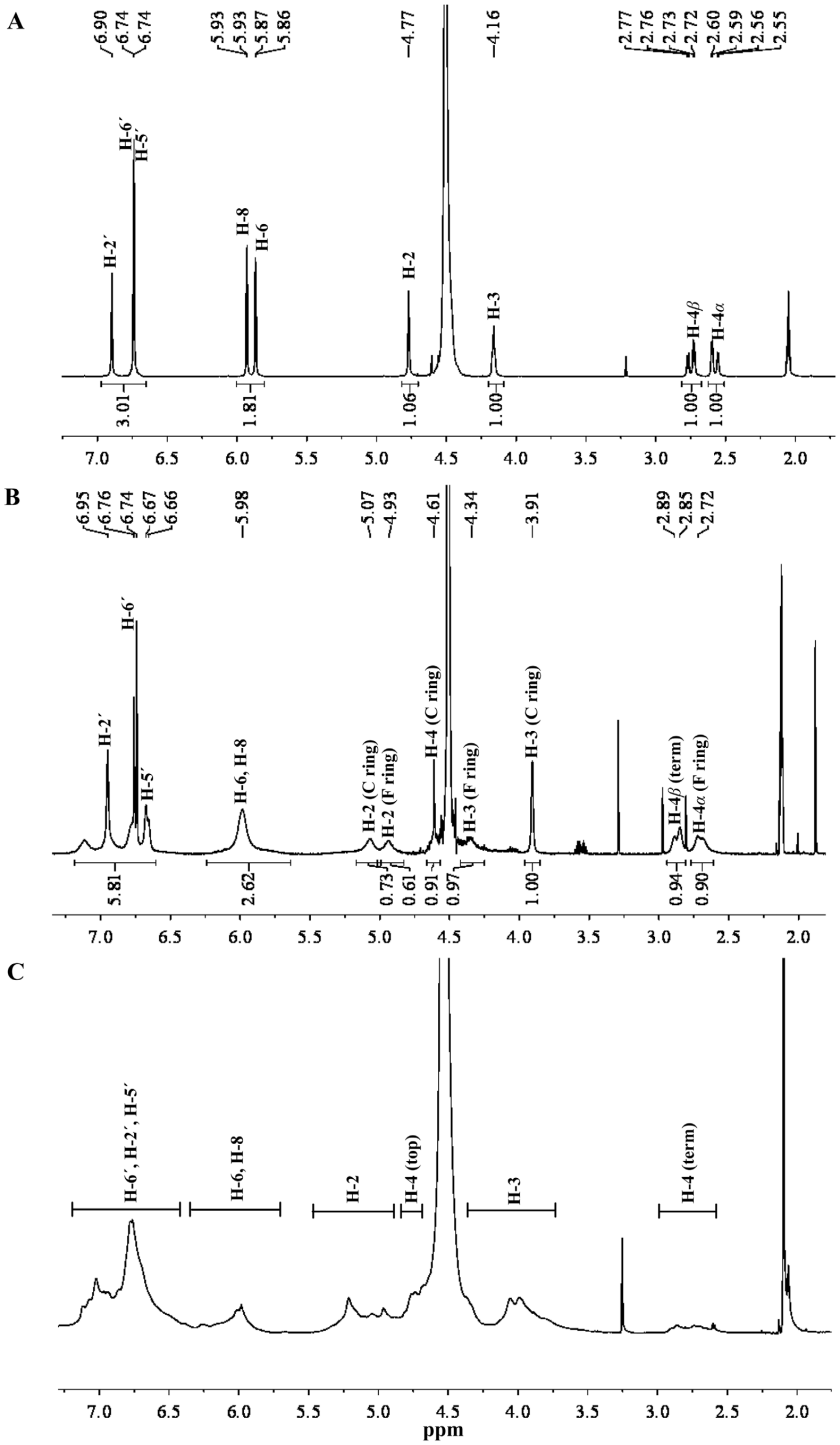
^1^H-NMR spectrum of EC (A), B2 (B) and mixed proanthocyanidins of root (C) in acetone-*d_6_*/D_2_O.

The compounds obtained from S1, S2, and S6 fractions were identified by ^1^H-NMR interpretation (Fig. 7). The results suggested that the compounds in S1 and S2 fractions were EC and procyanidin B2 (structures shown in Fig. 1), respectively, by comparing with the reference compounds in published literature^47^,^48^. Chemical shifts were expressed in ppm (*δ*) and were as follows. EC [^1^H NMR (400 MHz, acetone-*d*_6_) *δ*: 6.90 (s, 1H, H-2′), 6.74 (d, *J* = 1.0 Hz, 2H, H-5′ and H-6′), 5.93 (d, *J* = 2.3 Hz, 1H, H-8), 5.87 (d, *J* = 2.3 Hz, 1H, H-6), 4.77 (s, 1H, H-2), 4.16 (s, 1H, H-3), 2.75 (dd, *J* = 16.8, 4.5 Hz, 1H, H-4 *β*), 2.58 (dd, *J* = 16.8, 3.1 Hz, 1H, H-4*α*)]; procyanidin B2 [^1^H NMR (400 MHz, acetone-*d*_6_) *δ*: 6.95 (s, 1H, H-2′), 6.71 (dd, *J* = 34.1, 7.8 Hz, 2H, H-5′ and H-6′), 5.98 (s, 3H, H-6 and H-8), 5.07 (s, 1H, H-2 (C-ring)), 4.93 (s, 1H, H-2 (F-ring)), 4.61 (s, 1H, H-4 (C-ring)), 4.34 (s, 1H, H-3 (F-ring)), 3.91 (s, 1H, H-3 (C-ring)), 2.87 (d, *J* = 15.5 Hz, 1H, H-4 *β* (F-ring)), 2.72 (s, 1H, H-4*α* (F-ring))]. Compared with EC and the terminal unit (F-ring) of B2, the chemical shift of H-4 in top unit (C-ring, *δ* 4.61) of B2 was resonated to a lower field (Fig. 7A and 7B). H-2 and H-3 chemical shifts in the top unit (C-ring, *δ* 5.07 and 3.91) of B2 were also shifted, although the regularity was not as obvious as that of H-4 (Fig. 7B), which meant that the linkage was at position 4 in the C-ring.

S6 fraction was the polymer of mixed PAs. PAs with higher DP were difficult to purify due to their easy oxidation and isomerization. Compared with EC and B2 (Fig. 7A and 7B), the corresponding peaks of S6 (Fig. 7C) were broader, which is a feature of the polymers. In particular, the relative abundance of H-4 in terminal units and the significant decrease in H-6 and H-8 suggested the presence of C4–C6 or C4–C8 linkages of the polymeric PAs in the roots of tea plants.

Based on the ^1^H-NMR spectrum of EC, B2, and polymeric mixed PAs, coupled with the results of hydrolysis, MS, and ^13^C-NMR, it was concluded that the PAs of roots were homogeneous.

### Quantitative Determination of PAs in Roots of Tea Plants

Plant PAs are inherently complex with different linkages, diverse monomer units, and variable chain lengths^2^,^3^. The lack of appropriate standards and efficient analytical methods pose several challenges to the accurate quantitative determination of PAs. In the present study, we found the accumulation of PAs in tea roots, with a relatively simple structure (Fig. S2C, 5B, and 6B). Therefore, root PAs were the first to be analyzed quantitatively. After separation and purification, 1222 mg freeze-dried powders were obtained from 15 g dry-weight roots. It was estimated that the roots contained 8.1% of PAs. In addition, re-extraction and re-purification yielded 366.7 mg PAs from 400 mg of purified PAs, with a recovery rate of 91.7%. Therefore, purification could be accomplished for the quantitative estimation of PAs in roots, even though the technique was complicated. Spectrophotometry is a simple and effective method for the quantitative determination of PAs. In previous studies^44^,^49^,^50^, Vanillin-H_2_SO_4_ and DMACA-HCl methods were found to be more appropriate in the absence of interference by monomeric catechins. Furthermore, the butanol-HCl protocol was specifically used for the analysis of oligomeric and polymeric PAs, in the absence of anthocyanin and leucocyanidin in the samples^44^. Hence, a standard must be selected carefully, due to varying degrees of polymerization. For instance, in our study, epicatechin (monomeric PAs), B2 (dimeric PAs), and polymeric PAs (the mixture) exhibited different levels of sensitivity to Vanillin-H_2_SO_4_, DMACA-HCl, and butanol-HCl reagents (Fig. S4). Nevertheless, the results with different approaches varied even against identical standards. For example, when purified root PAs were used as standard, the PA content was 121.77 mg/g, 104.32 mg/g, and 85.06 mg/g by Vanillin-H_2_SO_4_, DMACA-HCl, and butanol-HCl protocols, respectively. The results of the butanol-HCl assay closely matched the results of the purification method. In addition, the accumulation of insoluble PAs was rare, and only a tenth of the soluble PAs were found in tea roots (data not shown).

### In Vitro Assay for the Auto-condensation of Flavan-3-ols

**Figure 8:**
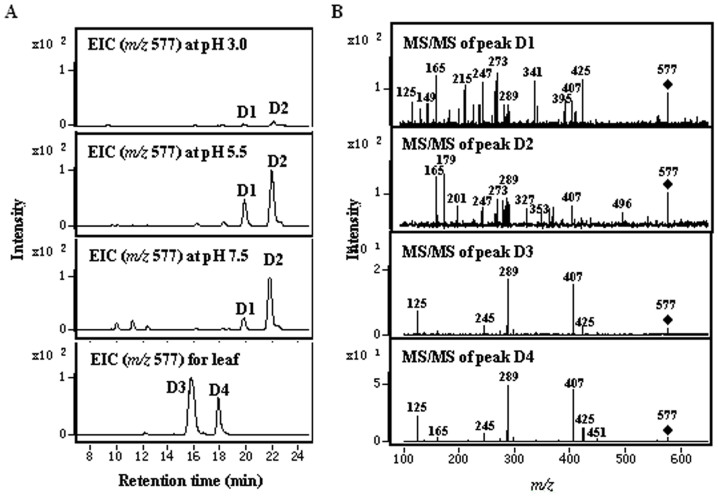
Analysis of the condensation products formed from EC in the negative mode. (A), Extraction ion chromatogram of dimers formed at different pH and extracted from leaves of tea plants; (B), MS/MS spectra of peak D1 (at 26 eV), and D2 (at 26 eV) obtained *in vitro*, and peak D3 (at 18 eV) and D4 (at 18 eV) extracted from the leaves of tea plants.

Non-enzymatic *in vitro* assay with different monomeric flavan-3-ols and procyanidin B2 was carried out for investigating the auto-condensation mechanism of PAs. Whether in acid or alkaline solution, EC could be condensed to form multiple dimers under mild conditions *in vitro* (25°C). In this study, the auto-condensation of EC showed pH-dependent and time-related features. Although the types of dimers of EC were consistent, their contents were different when assayed at different pH (Fig. 8A). However, the time-depent feature of auto-condensation was one of the most easily neglected aspects, since it was difficult to observe the process within a short time (less than 3 d) in mild reaction conditions (at pH 3.0), or in a protracted period of time (more than 2 d) due to the inevitable oxidation reaction (at pH 7.5).

As expected, the dimers formed *in vitro* did not match with those found in tea plants, in light of the retention time, fragments of MS/MS spectrum, and collision energy (Fig. 8A and 8B). For collision energy, a stronger energy (26 eV) was needed to collide the dimers of EC formed *in vitro* than that of procyanidin B2 (18 eV) in tea plants (Fig. 8B); thereby showing that the condensation pattern of the dimers formed *in vitro* was different from procyanidin B2 (4–8 linkage). It was speculated that the dimers formed from commercial EC *in vitro* were possibly the oxidation products, which did not exist in fresh tea plants.

**Figure 9:**
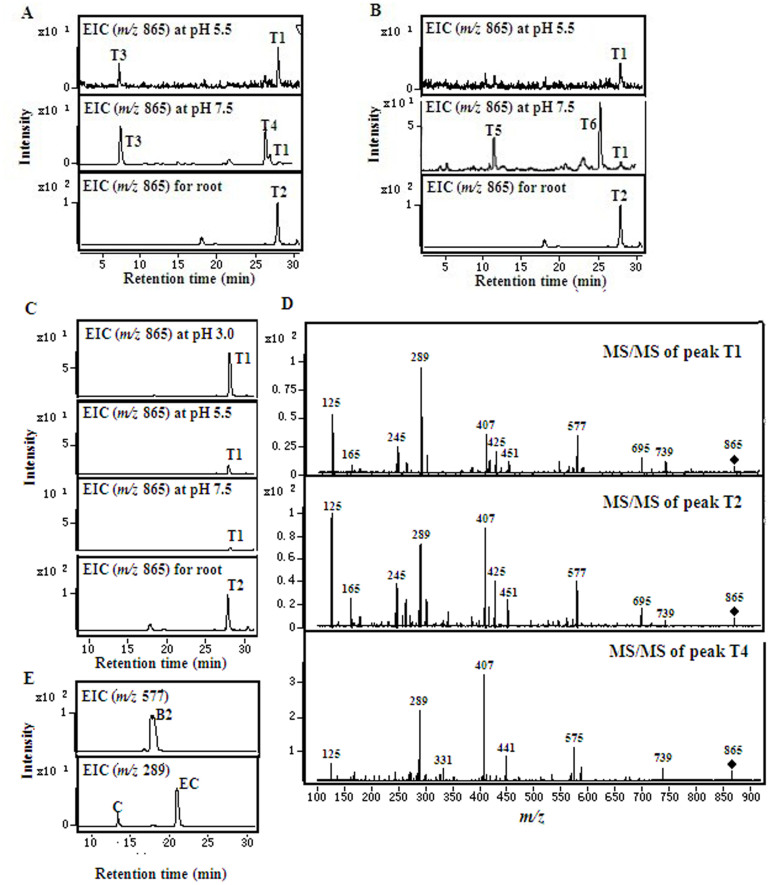
Analysis of the condensation products formed from either procyanidin B2 with EC or C, or procyanidin B2, in the negative mode. (A), Extraction ion chromatogram of trimers (*m/z* 865) formed from procyanidin B2 with EC at pH 5.5, pH 7.5, and extracted from roots of tea plants; (B), Extraction ion chromatogram of trimers (*m/z* 865) formed from procyanidin B2 with C at pH 5.5, pH 7.5, and extracts from roots of tea plants; (C), Extraction ion chromatogram of trimers (*m/z* 865) formed from B2 at different pH and extracted from roots of tea plants; (D), MS/MS of peak T1 (at 25 eV) and T4 (at 31 eV) obtained *in vitro*, and peak T2 (at 25 eV) extracted from the roots of tea plants. E, Extraction ion chromatogram of procyanidin B2 (*m/z* 577) and monomers (*m/z* 289) formed from procyanidin B2 at pH 3.0.

Moreover, the same trimers (*m/z* 865) were detected in the negative mode when a combination of procyanidin B2 and monomeric flavan-3-ols (EC or C), or procyanidin B2 alone was used as a substrate, in acid as well as alkaline solutions, but especially in the acid solution (Fig. 9). Based on the retention time and the fragments of MS/MS spectrum, the trimer obtained at peak T1 *in vitro* revealed the same compound as represented by peak T2 *in vivo* (Fig. 9A, 9B, 9C and 9D). This suggested that the trimer may have contained the C4–C8 or C4–C6 linkage formed from the direct reaction. In addition to the trimers, monomers (*m/z* 289) (Fig. 9E) and trace tetramers (*m/z* 1153) (data not shown) were also detected among the reaction products with procyanidin B2 as the substrate. This indicated that procyanidin B2 was degraded into its carbocation intermediate and monomeric flavan-3-ols; and the intermediate was further processed by nucleophilic addition of B2 to initiate the formation of trimers and tetramers. The trimers, T3 and T4, T5 and T6, were the major products formed at pH 7.5, which could essentially be the oxidation products of the substrates, when procyanidin B2 and EC or C were used as substrates, respectively (Fig. 9A and B).

Enzyme assays were designed to explore the polymerization mechanism of PAs *in vitro*, using crude enzymes extracted from tea plants and grape seeds, and commercial oxidases, including polyphenol oxidase (PPO) and peroxidase (POD). The reaction mixtures were observed to undergo oxidation when crude enzyme extracted from tea plants or the commercial oxidases (PPO and POD) were added. Peaks of *m/z* 575 and *m/z* 1151 were detected when procyanidin B2 was used along with the crude enzyme extracted from grape seeds (data not shown). This suggested the occurrence of an enzymatic oxidation reaction in the assays. However, no obvious oligomeric PAs were detected in weak acid solution (pH 5.5) or in alkaline solution (pH 7.5), when UPLC-QQQ-MS/MS was performed with procyanidin B2 and EC or only procyanidin B2 as the substrate (data not shown).

To sum up, the dimerization and trimerization reactions *in vitro* using monomeric flavan-3-ols as substrates could not reproduce the products formed *in vivo*. However, a trimerization reaction, which occurred *in vitro* with procyanidin B2 and monomeric flavan-3-ols (EC or C) or only procyanidin B2 as the substrate, was indicated to take place *in vivo* as well.

## Discussion

### The Qualitative and Quantitative Identification of PAs

Our results showed that LC-ESI-TOF-MS was particularly useful for the qualitative analysis of monomeric catechins and oligomeric PAs (DP < 3). UPLC-QQQ-MS/MS was used not only for qualitative analysis but also for quantitative analysis of the monomers and oligomers of PAs (DP < 4) based on the major fragment ions produced from MS/MS. MALDI-TOF-MS with precise molecular weights was found to be superior to NP-HPLC in terms of the qualitative analysis of oligomeric and polymeric PAs. The most important information for the structural characterization of PAs can be obtained from the ^13^C-NMR spectrum; the data include the stereochemistry of the heterocyclic ring at C2–C3 position, hydroxylation pattern of the B-ring, structures of the chain terminating units, and the DP of PAs^15^,^16^,^46^. In order to obtain the ^1^H-NMR spectrum, higher purity of the sample is required.

Our results additionally showed that the di- and tri-hydroxyl groups in the B-ring units, *cis*- and *trans*- C-2 units, galloylated and nongalloylated units of monomeric and polymeric flavan-3-ols co-existed in leaves. However, the tri-hydroxyl, *cis*- and galloylated flavan-3-ols were the main monomeric catechins units, and the (epi)catechin was the major unit of polymeric flavan-3-ols found, when the DP was greater than five. The PAs were observed to be abundant in roots, with the epicatechin unit acting as the predominant catechin of oligomeric and polymeric PAs. Comparative studies of flavonoid metabolism in tea leaves and roots will provide greater insights into the mechanisms of galloylation and polymerization. Indeed, the lack of galloylated catechins and compounds with tri-hydroxyl groups in the B-ring might be attributed to the absence of the expression of *CsF3'5'H* and *CsEGGT* and the related enzyme activity in roots^6^. It could be speculated from these results that the polymerization of the monomers was hampered by the 5'- position of hydroxyl groups in the B-ring, the galloyl group at the 3'- position of the C-ring, and the 2, 3-*trans* isomers. Roots were probably the ideal material for studying polymerization metabolism, due to the presence of di-hydroxyl groups in the B-ring and lack of other flavonoids.

The major limitation to accurate quantitation of PAs is the lack of availability of appropriate standards. Spectrophotometry staining methods with Vanillin-H_2_SO_4_^44^, or DMACA-HCl reagents^49^ are the most commonly used quantitative techniques. Purified PAs from sainfoin (*Onobrychis* *Viciifolia*) were used by Li et al. as a standard to estimate the threshold PA content for bloat safety in forage legumes^49^. Zhang et al. and Lin et al. utilized purified PA from *Lithocarpus glaber* leaves as a standard to estimate the concentration of PAs by the butanol-HCl method^16^.

PAs from plant sources are naturally complex due to the occurrence of several kinds of monomer units, different types of linkages, and variable chain lengths^1^,^2^,^3^,^5^,^15^,^16^. Further, the structural characterization of PAs in each of our samples was found to be varying, requiring confirmation with different standards. Compared with the relatively simple and unitary PAs in tea roots, the existence of a plethora of monomeric catechins and impurities in tea leaves complicates the purification method for quantitative analysis, rendering it impracticable. The spectrophotometry protocol entails staining with Vanillin-H_2_SO_4_^44^, and DMACA-HCl^44^,^49^ reagents, which react with the A-ring of catechins. It is the most common quantitative analytical method for PAs. In hot mineral acid solutions, PAs get converted into anthocyanidins, enabling quantitative analysis and determination of absorbance at 550 nm A_550_. However, the spectrophotometry protocols were found to be affected by a series of factors^44^,^51^, with undesirable effects on their stability and reproducibility.

### The Auto-Condensation Mechanism of PAs

According to a former study^52^, all the rings (A, B, and C) of the flavonoid framework are reactive and can undergo three specific types of reactions. The A ring, with a meta-hydroxy substitution pattern, is prone to the polycondensation reaction. The B ring, with an ortho-dihydroxy substitution pattern, leads to the oxidation reaction. The C ring, when positively charged in an acid solution, is inclined to process direct reactions, such as proanthocyanidins condensation.

The condensation mechanism of proanthocyanidins has gained much attention since the 1970s in the last century. Based on the structure and stereochemistry of proanthocyanidins, the isotopic tracer studies on procyanidin biosynthesis, and the biogenetically patterned synthesis of natural procyanidins in the laboratory, it was hypothesized that the dimers are biosynthesized from two metabolically distinct units, the nucleophilic flavan-3-ols (constituting the lower unit), and the electrophilic C-4 carbocation (forming the upper unit), which may generate from flav-3-en-3-ol^17^,^53^,^54^,^55^,^56^,^57^.

The origin of C-4 carbocations or flav-3-en-3-ol *in vivo* is intriguing, and these compounds may be difficult to identify directly in plants; which in turn renders it difficult to comprehend the condensation mechanism of proanthocyanidins. However, since 1980, several progresses have been made in the biochemical and molecular fields for the understanding of flavonoid biosynthesis. Stafford et al. reported that the dihydroflavonols (dihydroquercetin and dihydromyricetin) could sequentially be reduced to form 3,4-diol and 2,3-*trans*-(–)flavan-3-ols by two NADPH-dependent reductases, which were later reported to be DFR^58^ and LAR^59^ derived from tissue cultures of *Douglas fir* and *Ginkgo biloba*^60^,^61^. Meanwhile, they also found that the production of gallocatechin (4a-8)-catechin dimer could be detected when (+)-catechin was added to the enzymic incubation mixture with dihydromyricetin in acidic medium, suggesting that the carbocation or quinone methide intermediate might be derived from 3,4-diol (leucodelphinidin) and subsequently added to an electrophilic group, such as (+)-catechin to form dimer with 4–8 or 4–6 bond linkages^62^. Additionally, they theorized the presence of a C3-epimerase involved in the biosynthesis of 2,3-*cis* (–)flavan-3-ols^63^. However, it was later demonstrated by Xie et al. that the *BANYULS* (*BAN*) genes from *Arabidopsis thaliana* and *Medicago truncatula* could encode anthocyanidin reductase, which converted cyanidin to 2,3-*cis*-(–)-epicatechin^64^; and therefore, speculated that flav-3-en-3-ol might be an intermediate in this conversion process^65^. Further, Dixon et al.^19^ provided a theoretical view that the carbocation and quinone methide intermediates might be derived from the corresponding flavylium ion of anthocyanidins in acidic conditions catalyzed by polyphenol oxidase (PPO). They alternatively proposed that EC or C could be converted to carbocation derivatives via corresponding quinones and flav-3-en-ols, which were in turn catalyzed by PPO or through non-enzymatic coupled oxidation reactions. Certain studies have shown that tyrosinase, PPO, and laccase-like enzymes might be involved in the enzymatic condensation and polymerization of PAs^31^,^32^,^33^.

Based on the aforementioned studies, leucoanthocyanidin, anthocyanidin, and flavan-3-ols were likely to be the substrates in the formation of C-4 carbocation or flav-3-en-3-ol. One of the reasons for the insufficient knowledge on the condensation mechanism of proanthocyanidins is the lack of a biogenetically patterned synthesis assay. For instance, the suitable substrates, leucoanthocyanidin and carbocation, are commercially unavailable, and the flavan-3-ols have been found to be unstable and readily oxidized *in vitro*. Considering the higher content of PAs in older stems and roots, and the irrelevant trend between the accumulation of PAs and the relative expression rate of LAR and ANR genes in tea plants^6^, we inferred that the condensation and polymerization of PAs occur only after the biosynthesis of monomeric flavan-3-ols.

In the present study, procyanidin B2 could not be detected, as expected, when only EC was used as a substrate in the non-enzymatic *in vitro* assay in acid or alkaline solution (from pH 3.0 to 7.5) (Fig. 8). Therefore, it was proved that the carbocation or flav-3-en-3-ol did not generate from EC. The primary dimers in the above-mentioned assay needed higher energy to collide as compared to the dimers with C4–C8 or C4–C6 linkages, such as procyanidin B2 (Fig. 8A and 8B); and thus, the generation of anthocyanin became difficult when submitted to acid-catalyzed hydrolysis. These characteristics were consistent with that of certain dimers with biphenyl linkages (φB–φA) and biphenyl ether linkages (φB-O-φA), which usually form from the oxidation reactions of flavanols^52^.

The same procyanidin trimers could be detected in the extracts of tea plants as well as in the non-enzymatic *in vitro* assay, when procyanidin B2 with EC or C were used as a substrate in acid and alkaline solutions, respectively (Fig. 9A and 9B). Interestingly, the same procyanidin trimer was also detected when only procyanidin B2 was used as the substrate in non-enzymatic *in vitro* assay (Fig. 9C). This suggested that procyanidin B2 not only released the carbocation as electrophilic upper units, as expected, but also as nucleophilic lower units, to form the procyanidin trimer *in vitro* or *in vivo*.

**Figure 10:**
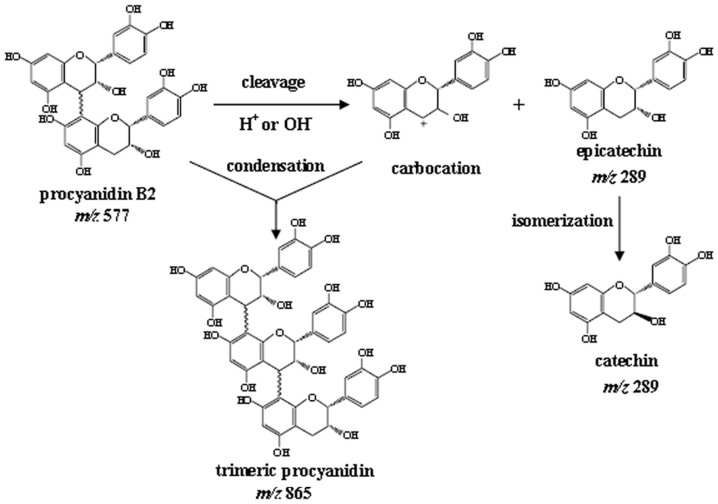
Speculative scheme for the mechanism of auto-condensation of proanthocyanidins.

These findings led us to speculate that the mechanism of auto-condensation involves the release of carbocations through certain cleavage reactions of B2; following which, the carbocations as electrophiles were involved in the direct reactions with procyanidin B2 as nucleophiles, leading to the formation of a trimer *in vitro* or *in vivo*, as found in the roots of tea plants (Fig. 10).

Nevertheless, the procyanidin trimerization in the non-enzymatic *in vitro* assay showed a very low conversion rate, approximately 5–10%, which did not correspond to the high concentration of PAs in tea plants. Whether or not a more efficient reaction in tea plants occurs, such as enzymatic condensation, still needs to be probed. In order to research the enzymatic condensation mechanism of PAs, we worked with procyanidin B2 as a substrate in weak acid (pH 5.5) as well as alkaline solutions (pH 7.5); however, no enzymatic product was detected. Further studies could, therefore, prove instrumental for the in-depth understanding of the mechanisms of proanthocyanidins condensation, auto- or enzymatic condensation.

## Methods

### Plant Materials

The samples of leaves, stems and roots were collected from mature tea plants during early summer from the Experimental Tea Garden, Anhui Agricultural University, Anhui, China. The collected samples were immediately frozen in liquid nitrogen and stored at −30°C prior to analysis.

### Chemicals and reagents

The compounds viz., catechin, epicatechin, gallocatechin, epigallocatechin, epigallocatechin gallate, epicatechin gallate, and procyanidin B2 were obtained from Sigma (St Louis, MO, USA). Cyanidin chloride and delphinidin chloride were purchased from Axxora Co. Ltd. (Lausanne, Switzerland). Benzyl mercaptan was obtained from Aladdin (Shanghai, China). HPLC grade methanol, acetonitrile, chloroform, dichloromethane, ethyl acetate, *n*-hexane, *n*-butanol, and acetic acid were procured from Tedia Co. Ltd. (Fairfield, OH, USA). The following materials and equipment were used for separation and chromatography: Sephadex LH-20 (GE Healthcare, Sweden); silica GF254 TLC sheet (5 × 20 cm; HeFei BoMei Biotechnology Co., Hefei, China); ordinary-phase silica gel column (silica gel H, 200–300 mesh; Anhui Liangchen Silicon Material Co. Ltd., Huoshan, China); and a Varian Prostar HPLC instrument, Model 325 (Varian, Mulgrave, Australia). ^1^H-NMR and ^13^C-NMR spectra were obtained on an AVANCE AV 400 (400/100 MHz) spectrometer (Bruker, Fallanden, Switzerland). ^13^C-NMR spectra were recorded at 150 MHz in acetone-*d6*/D2O mixture using a Varian Mercury-600 spectrometer (USA).

### Extraction and Purification of PAs

PAs were extracted and purified according to the method described by Gu et al^1^ with some modifications. Samples (leaves, stems, or roots; 120 g) were extracted three times with an extraction solvent containing 70% acetone and 0.5% acetic acid. The extracts were combined, and the acetone was evaporated at 30°C using a rotary evaporator under vacuum. The resultant brown slurry (approximately 600 mL) was extracted three times with chloroform (500 mL each) and the aqueous fraction was applied to a 50 × 3.5 cm Sephadex LH-20 column. The column was eluted with 50% methanol, followed by elution with 70% acetone to remove all the sugars and phenolic acids. The brown acetone fraction containing PAs was collected under partial vacuum at 30°C, and then freeze-dried to obtain a fluffy rough PAs powder (from individual sources of leaves, stems, and roots), which was subsequently characterized using RP-HPLC-TOF-MS, UPLC-QQQ-MS/MS, NP-HPLC, MALDI-TOF-MS, and ^13^C-NMR analyses.

The rough PAs powder extracted from the roots was further purified on thin-layer chromatography (TLC, GF254 silica gel plate) on a small scale, with elution using chloroform: methanol: formic acid (28:10:1, v/v)^66^, or on a larger scale on a silica gel H column chromatography (50 × 3.5 cm) with elution using chloroform: methanol: formic acid (7:3:0.4, v/v). The PAs fractions detected qualitatively by the Vanillin-H_2_SO_4_^44^, and DMACA-HCl reagent^49^ were collected and freeze-dried. The S1, S2, and S6 fractions were further purified by preparative HPLC. The purified fractions were concentrated and freeze-dried for ^1^H-NMR analysis of the characteristics of PAs in roots.

### Identification of PAs in Tea Plants

#### Reversed-Phase LC-ESI-MS

Liquid chromatography (LC)-time of flight (TOF) mass spectrometer (MS) system and the ultra performance liquid chromatography (UPLC)-triple quadrupole mass spectrometry (QQQ-MS/MS) system were used for the qualitative identification of monomeric catechins and oligomeric PAs as described by Jiang et al.^6^

#### Hydrolysis

Upon treatment with hot mineral acid solutions, PAs were depolymerized into colored anthocyanidins with absorbance maxima around 550 nm^44^,^51^. PAs of tea plants were assayed by the butanol-HCl method according to Jiang et al.^6^. The hydrolysis products (anthocyanidins) were further characterized using reversed-phase (RP)-HPLC analysis.

#### Thiolysis

We performed thiolysis of PAs based on the methods described by Guyot et al.^13^ and Gu et al.^1^. Briefly, in a 250-μL polypropylene insert (Fisher Scientific, Boston, MA), 50 μL solution of each fraction (2 mg/mL in methanol) was mixed together with 50 μL of methanol acidified with concentrated HCl (3.3%, v/v) and 100 μL of benzyl mercaptan (5%, v/v in methanol). The inserts were transferred into a 1.5-mL vial and sealed with an inert Teflon cap (Agilent Technologies, Wilmington, DE). The reaction was carried out at 40°C for 30 min and then kept at room temperature for 10 h to ensure complete degradation. To minimize further epimerization and any side reaction, the reaction mixtures were left in the freezer (−20°C) until 5 μL was injected directly for RP-HPLC analysis, for additional characterization of the products of thiolysis (catechins and their benzylthioethers).

#### Reversed-Phase HPLC Analysis of Products of Hydrolysis and Thiolysis

A Phenomenex Synergi 4 u Fusion-RP80 column (particle size: 5 μm, length: 250 mm, and internal diameter: 4.6 mm) was used at a flow rate of 1.0 mL min^−1^. The column oven temperature was set at 25°C. The mobile phase consisted of 1% acetic acid in water and 100% acetonitrile. The gradient of the hydrolyzed products increased linearly from 13 to 30% (v/v) at 30 min, to 13% at 32 min. The DAD was set at 280 nm and 530 nm, respectively for real-time monitoring of the peak intensities. The gradient of the products of thiolysis increased linearly from 13 to 24.3% (v/v) at 20 min, to 40% (v/v) at 30 min, to 55% (v/v) at 45 min, to 80% at 55 min, to 13% at 60 min. The DAD was set at 280 nm for catechin, epicatechin, gallocatechin, epigallocatechin, epigallocatechin gallate, epicatechin gallate, and their benzylthioethers.

#### Normal-Phase HPLC Analysis

The analysis was conducted using an Agilent 1100 HPLC system with Phenomenex Luna 5 u Silica (2) 100 A column (particle size: 5 μm, length: 250 mm, and internal diameter: 4.6 mm). The detection wavelength was set at 280 nm and the column oven temperature at 25°C. The ternary mobile phase consisted of (A) methanol, (B) dichloromethane, and (C) acetic acid and water (1:1 v/v). The 57-min linear gradient based on that of Hammerstone et al.^67^ with some modifications was as follows: 0–35 min, 14.0–30% A; 35–40 min, 30–32% A; 40–45 min, 32–86.0% A; 45–52 min, 86.0% isocratic; 52–57 min 86.0–14.0% A, followed by 10 min of reequilibration of the column. A constant of 2.0% C was kept throughout the gradient.

#### MALDI-TOF-MS Analysis

MALDI-TOF-MS was performed according to the method described by Zhang et al.^16^. The MALDI-TOF-MS spectra were recorded on a Bruker Reflex III instrument (Germany). The irradiation source was a pulsed nitrogen laser with a wavelength of 337 nm, and the duration of the laser pulse was 3 ns. In the positive reflection mode, an accelerating voltage of 20.0 kV and a reflectron voltage of 23.0 kV were used. Spectra of PAs were obtained from a sum of 100–150 shots and calibrated using angiotensin II (1,046.5 MW), bombesin (1,619.8 MW), ACTHclip18–39 (2,465.2 MW), and somatostatin 28 (3,147.47 MW) as external standards. 2,5- dihydroxybenzoic acid (DHB, 10 mg/mL aqueous solution) was used as the matrix. The sample solutions (7.5 mg/mL aqueous) were mixed with the matrix solution at a volumetric ratio of 1:3. The mixture (1 μL) was applied to the steel target. Amberlite IRP-64 cation-exchange resin (Sigma-Aldrich), equilibrated in deionized water, was used to deionize the analyte/matrix solution three times. Cesium chloride (1 mg/mL) was mixed with the analyte/matrix solution at 1:3 volumetric ratio to promote the formation of a single type of ion adduct ([M + Cs]^+^)^68^,^69^.

#### NMR Analysis

A protocol described by Kraus et al.^15^ was employed for nuclear magnetic resonance (NMR) spectroscopy analysis. The preliminary purified PAs of leaves and roots were analyzed by ^13^C-NMR and the purified PAs of roots were identified by ^1^H-NMR. The compounds were dissolved in a mixture of 0.25 ml acetone and 0.25 ml D_2_O and transferred through sintered glass filters into 5-mm-diameter NMR tubes. ^1^H-NMR and ^13^C-NMR spectra were obtained on an AVANCE AV 400 (400/100 MHz) spectrometer (Bruker, Fallanden, Switzerland), using inverse-gated decoupling, 45° pulse, 0.4 sec acquisition time, 2.6 sec delay, and 25,000–30,000 scans.

### Extraction and Quantitative Determination of PAs in Tea Roots

The total PAs were extracted and purified from 50 g of roots. The extraction and preliminary purification were carried out according to the above described protocols. The freeze-dried fluffy powders were weighed to calculate the percentage of PAs. We selected 400 mg of powdered root PA with purity greater than 90%, for re-extraction and purification by Sephadex LH-20 column to measure the recovery rate of purified PAs.

Quantitative determination of PAs in root was accomplished using the Vanillin-H_2_SO_4,_^44^, DMACA-HCl^49^, and butanol-HCl^6^ methods; EC, B2, and purified PAs of root were used as standards, respectively.

### Auto-condensation of Flavan-3-ol *in Vitro*

In acid solution, the reaction mixture was incubated at 25°C for 15 d, in a total volume of 0.2 mL consisting of 100 mM acetate buffer (pH 3.0), or 100 mM Mes buffer (pH 5.5), and 0.4 mM each of procyanidin B2, EC, or C. In alkaline solution, the reaction mixture were determined at 25°C with 24 h incubations, in a final volume of 0.2 mL consisting of 100 mM Tris-HCl buffer (pH 7.5), and 0.4 mM of the flavan-3-ols, as used in acid solution. The reaction products were then analyzed by UPLC-QQQ-MS/MS system as described by Jiang et al.^6^. The extraction of crude enzyme was carried out as described by Liu et al.^7^, and the reaction mixture was similar to to that of a non-enzymatic reaction.

## Author Contributions

T.X., L.P.G., X.L.J., Y.J.L. and Y.S.W. conceived and designed the experiments. X.L.J., Y.J.L., Y.H.W., H.R.T., Y.M.Q. and F.M. performed the experiments. X.L.J., M.Z.L., L.Z., L.L. and Y.J.L. conducted the theoretical analysis. L.P.G., X.L.J., T.X. and Y.J.L. co-wrote the manuscript. All authors reviewed the manuscript. X.L.J. and Y.J.L. contributed equally to this work.

## Supplementary Material

Supplementary Information supplementary Information

## Figures and Tables

**Table 1:** Analysis of monomeric catechins and oligomeric proanthocyanidins in leaf, stem and root of tea plant

Peak/no.	t_R_ (min)	[M – H]^+^/[M - H]^−^ (*m/z*)	MS/MS (*m/z*)	UV λmax (nm)	identification	Leaf	Stem	Root
1	11.060	−/609.11332	−/125,305,423	272	GC-GC	+	+	—
2	14.166	−/305.06385	−/125, 165,179,219	275	gallocatechin^a^	+	+	—
3	16.292	−/593.15606	−/289,305, 407,423	270	C-GC	+	+	—
4	16.765	−/425.03299	−/169,273	274	epiafzelechin gallate	+	+	—
5	18.587	−/305.06517	−/125,137,179, 219	275	epigallocatechin^a^	+	+	—
6	20.343	−/761.12183	−/125,305,423,591	275	EGC-EGCG	+	+	—
7	20.630	−/289.06836	−/109,125,203,245	278	catechin^a^	+	+	—
8	23.668	−/577.12389	−/125,289,407,425,451	273	EC-EC dimer	+	+	—
9	25.018	−/577.12284	−/125,289,407,425,451	273	Procyanidin B2^a^	+	+	++
10	26.621	−/745.12819	−/169,407,423,593	278	ECG-EGC	+	+	—
11	27.330	−/289.07148	−/109,125,203,245	278	epicatechin^a^	+	+	+
12	30.993	−/457.09664	−/125,169,305	278	epigallocatechin gallate^a^	+	+	—
13	34.908	−/865.18022	−/125,407	278	EC-EC-EC PAs trimer	+	+	++
14	35.280	−/729.13377	−/125,245,407,577	278	EC-ECG	+	+	—
15	35.823	−/897.15219	−/−	278	ECG-EGCG or EGC-EGC-EC	+	+	—
16	36.596	−/273.07432	−/97,229	275	epiafzelechin	+	+	—
17	38.132	−/729.17308	−/289,407, 441,559	278	EC-ECG	+	+	—
18	50.200	−/441.10108	−/125,169, 271,289	278	epicatechin gallate^a^	+	+	—
19	52.896	−/1153	−/−	278	tetrameric procyanidin	—	—	+
20	57.201	−/1441	−/−	278	pentameric procyanidin	—	—	+

Notes: ^a^Compounds positively identified by direct comparison with the standard; other compounds were approximately identified qualitatively by comparing the wavelengths of maximum absorbance, protonated and deprotonated molecules ([M - H]^+^/[M - H]^-^), and fragment ions (PI/NI) from the published literature; t_R_ (min) was the retention time in Figure 2. [M - H]^+^/[M - H]^-^ (*m/z*) of compounds were detected by LC-TOF-MS; MS/MS (*m/z*) of compounds were detected by UPLC-TOF-MS/MS. —, not detected; +, detected; ++, signal was stronger.

**Table 2:** Thiolysis analysis of proanthocyanidins in leaf, stem and root of tea plant

Organ	DP value
Annual leaf	1.03 ± 0.02
Biennial leaf	1.02 ± 0.03
Annual stem	1.35 ± 0.04
Biennial stem	3.13 ± 0.08
Fibrous root	5.85 ± 0.05
Taproot	5.41 ± 0.11

Notes: DP, degree of polymerization; the mean ± SD of three independent analyses.

**Table 3:** MALDI-TOF-MS analysis of proanthocyanidins in leaf and root of tea plant

DP	Composition			Interflavan bond		Observed [M + Cs]^+^		Calculated [M + Cs]^+^
	di	tri	gallate	A Type	B Type	Leaf	Root	
3	3	0	0	0	2	999.1504	999.3355	999
	3	0	0	2	0	995.1264	—	995
	2	1	0	0	2	1015.1246	—	1015
	2	1	0	2	0	1011.0491	—	1011
	1	2	0	0	2	1031.1058	—	1031
	0	3	0	0	2	1047.1108	—	1047
	3	0	1	0	2	1151.1466	—	1151
	3	0	1	2	0	1147.0060	—	1147
	2	1	1	0	2	1167.1640	—	1167
	2	1	1	2	0	1163.0302	—	1163
	0	3	1	0	2	1183.1965	—	1183
	0	3	1	1	1	1181.0724	—	1181
	0	3	1	2	0	1179.0103	—	1179
4	4	0	0	0	3	1287.2284	1287.4182	1287
	3	1	0	0	3	1303.2343	—	1303
	2	2	0	0	3	1319.1621	—	1319
	1	3	0	0	3	1335.1359	—	1335
	0	4	0	0	3	1351.1807	—	1351
	4	0	1	0	3	1439.2525	—	1439
	3	1	1	0	3	1455.3804	—	1455
	2	2	1	0	3	1471.2207	—	1471
	1	3	1	0	3	1487.1755	—	1487
5	5	0	0	0	4	1575.2790	1575.5575	1575
	4	1	0	0	4	1591.2459	—	1591
	5	0	1	0	4	1727.3015	—	1727
6	6	0	0	0	5	1863.3315	1863.6723	1863
	5	1	0	0	5	1879.3359	—	1879
	4	2	0	0	5	1895.3481	—	1895
	6	0	1	0	5	2015.9421	—	2015
7	7	0	0	0	6	2152.3955	2151.7832	2151
	6	1	0	0	6	2167.4018	—	2167
8	8	0	0	0	7	2440.4849	2439.8506	2439
9	9	0	0	0	8	2728.4940	2727.9653	2727
10	10	0	0	0	9	—	3016.0829	3016
11	11	0	0	0	10	—	3305.0805	3305
12	12	0	0	0	11	—	3593.3321	3593

Notes: di, di- hydroxyl groups in the B-ring; tri, tri- hydroxyl groups in the B-ring; gallate, gallate groups in the C-ring at C3.
