# Supplementary material for: Analysis of accumulation patterns and preliminary study on the condensation mechanism of proanthocyanidins in the tea plant [Camellia sinensis]
Source: Sci Rep. 2015 Mar 4;5:8742. doi: 10.1038/srep08742 (PMC4348662; doi:10.1038/srep08742)
Supplement: Supplementary Information [file srep08742-s1.pdf]

Analysis of accumulation patterns and preliminary study on the condensation mechanism of proanthocyanidins in the tea plant [*Camellia sinensis*]

Xiaolan Jiang[a1], Yajun Liu[b1], Yahui Wu[a] ,Huarong Tan[c], Fei Meng[a], Yun sheng Wang[b], Mingzhuo Li[a], Lei Zhao[d], Li Liu[a], Yumei Qian[a], Liping Gao[b]*, and Tao Xia[a]*

[a]Key Laboratory of Tea Biochemistry and Biotechnology, Ministry of Education in China, Anhui Agricultural University, Hefei, Anhui, China

[b]School of Life Science, Anhui Agricultural University, Hefei, Anhui, China

[c]Biotechnology Center, Anhui Agricultural University, Hefei, Anhui, China

[d]College of Horticulture, Qingdao Agricultural University, Qingdao, Shandong, China

*Corresponding author; e-mail, xiatao62@126.com; gaolp62@126.com

[1] These authors contributed equally to this work.

Corresponding author:

Tao Xia

Key Laboratory of Tea Biochemistry and Biotechnology, Ministry of Education in China, Anhui Agricultural University, 130 West Changjiang Rd, Hefei, Anhui 230036, China

Tel: 86-551-5786003, Fax: 86-551-5785729, E-mail: xiatao62@126.com;

Liping Gao

School of Life Science, Anhui Agricultural University, 130 West Changjiang Rd, Hefei, Anhui 230036, China

Tel: 86-551-5786129, Fax: 86-551-5785729, E-mail: gaolp62@126.com

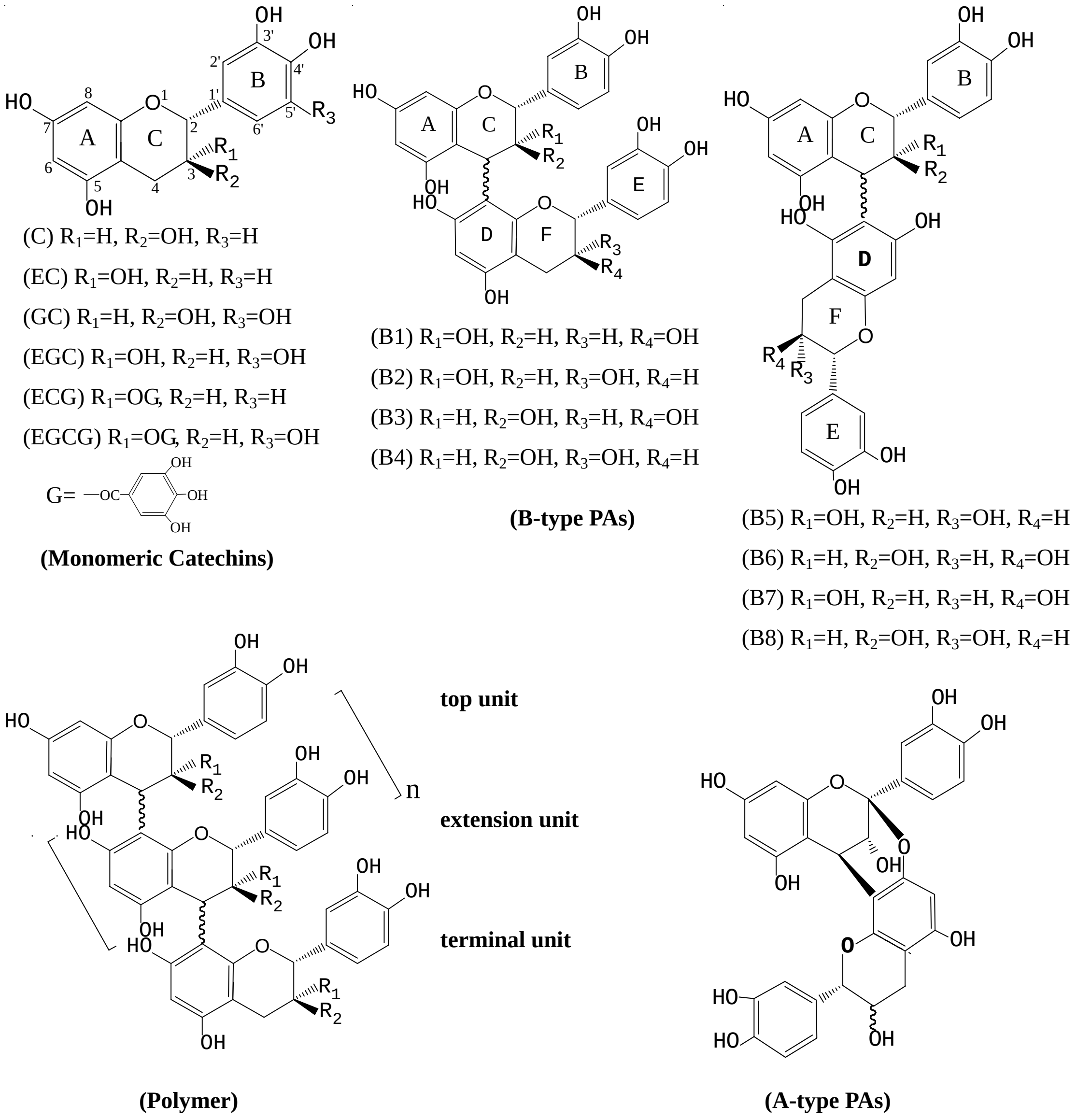

Figure S1. Structures of proanthocyanidin monomers, oligomers and polymers. Monomers: C, catechin; EC, epicatechin; GC, gallocatechin; EGC, epigallocatechin; ECG, epicatechin gallate; EGCG, epigallocatechin gallate; Oligomers: B-type PAs, B-type proanthocyanidins; B1~B4, dimeric proanthocyanidins with C4-C8 bond; B5~B8, dimeric proanthocyanidins with C4-C6 bond; A-type PAs, A-type proanthocyanidins; Polymer: polymeric proanthocyanidins.

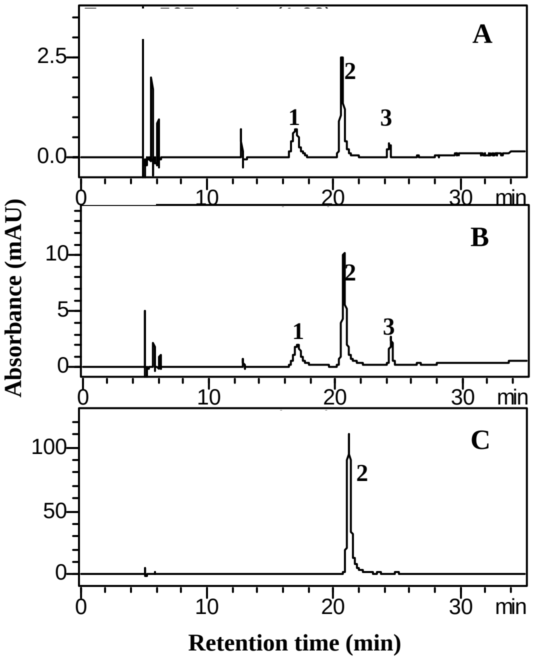

Figure S2. RP-HPLC analysis of anthocyanidin by butanol-HCl hydrolysis from leaf (A), stem (B) and root (C) extracts of tea plants. Peaks 1, 2, and 3 represent delphinidin, cyanidin, and pelargonidin, respectively.

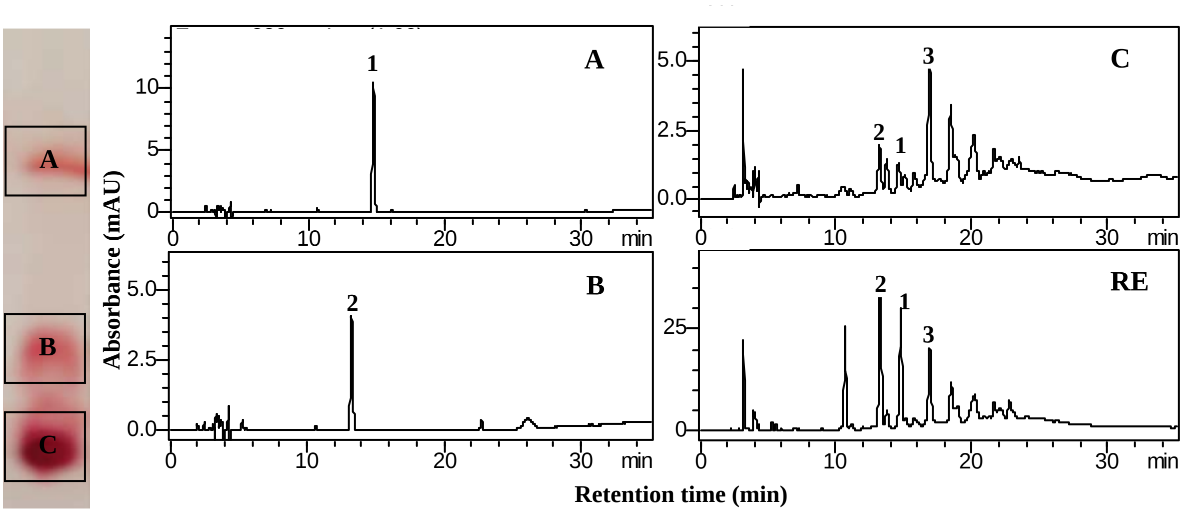

Figure S3. TLC (left) and HPLC (middle and right) chromatograms of catechins and proanthocyanidins in root extracts. A, B, and C, represent the HPLC analyses of spot A, spot B, and spot C, respectively, as purified by TLC; RE, HPLC analysis of root extract. 1, epicatechin; 2, dimeric procyanidin; 3, trimeric procyanidin.

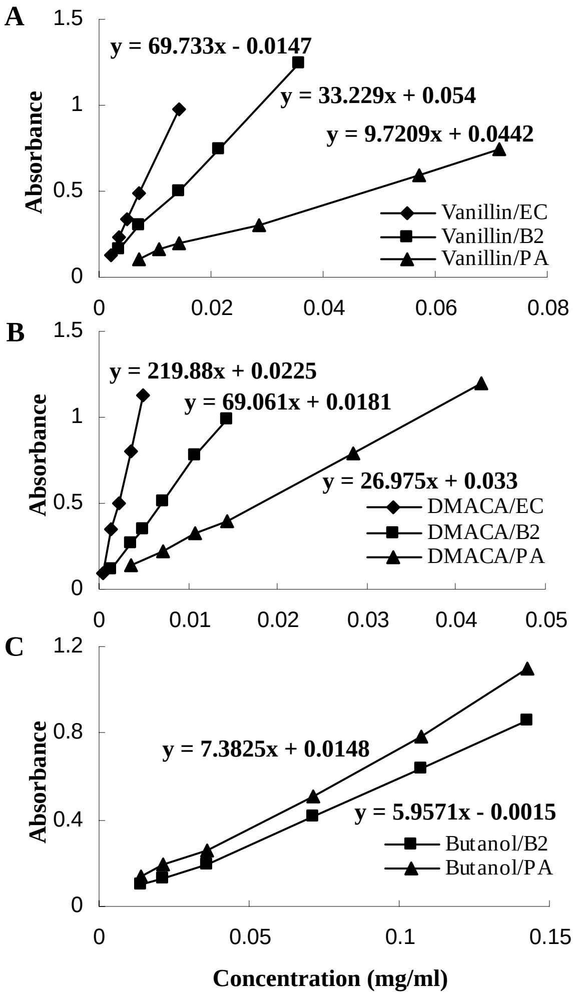

Figure S4. Responses of flavan-3-ols to different acidic reagents. A, vanillin-H2SO4; B, DMACA-HCl; and C, butanol-HCl.
